# Total arch replacement with the frozen elephant trunk technique for a right-sided aortic arch with a Kommerell diverticulum and aberrant right vertebral artery

**DOI:** 10.1093/icvts/ivac075

**Published:** 2022-03-25

**Authors:** Tomoki Nagata, Shinichi Iwakoshi, Takashi Yamada, Hiroyuki Johno

**Affiliations:** 1 Department of Cardiovascular Surgery, Takaishi Fujii Cardiovascular Hospital, 2-15-18 Ayazono, Takaishi-shi, Osaka, 592-0014, Japan; 2 Department of Radiology, Nara Medical University, 840 Shijo, Kashihara-shi, Nara, 634-8521, Japan; 3 Department of Cardiology, Takaishi Fujii Cardiovascular Hospital, 2-15-18 Ayazono, Takaishi-shi, Osaka, 592-0014, Japan

**Keywords:** right sided aortic arch, Kommerell diverticulum, aberrant vertebral artery, frozen elephant trunk

## Abstract

The best treatment for a right-sided aortic arch (RAA) and Kommerell diverticulum (KD) has not been determined due to the rarity of these conditions. The current trend in the treatment of this disease is to increase the endovascular approach without a sternotomy. We describe a rare condition with an association of an RAA with a KD of an aberrant left subclavian artery and an anomalous right vertebral artery originating from the aortic arch (AVA). The left vertebral artery was missing. Also, there was an incomplete circle of Willis due to the absence of the left and right posterior communication arteries. Therefore, the AVA was the only artery to supply the vertebral-basilar system. In our case, a simple thoracic endovascular aortic repair was not suitable because of the sharply curved arch and short landing zone. Also, a debranching thoracic endovascular aortic repair was not appropriate because that approach would not permit reconstruction of the AVA. The patient successfully underwent a total arch replacement with the frozen elephant trunk technique. This procedure could be an effective option for patients with RAAs with KDs associated with another arch vessel anomaly.

## INTRODUCTION

An aberrant left subclavian artery (ALSA) with a right-sided aortic arch (RAA) is a rare aortic arch anomaly. Aneurysm formation usually occurs at the origin of the ALSA, which is known as a Kommerell diverticulum (KD). A KD has a high risk of an aortic dissection or rupture. Symptoms of oesophageal or tracheal compression may develop. Surgical, endovascular or hybrid procedures have been described to treat RAAs and KDs [1–3]. This case report describes a rare condition with the association of an RAA with a KD of an ALSA and an anomalous right vertebral artery originating from the aortic arch (AVA), which was successfully treated by an open surgical repair using a total arch replacement (TAR) with a frozen elephant trunk technique (FET) procedure.

## CASE PRESENTATION

**Figure 1:**
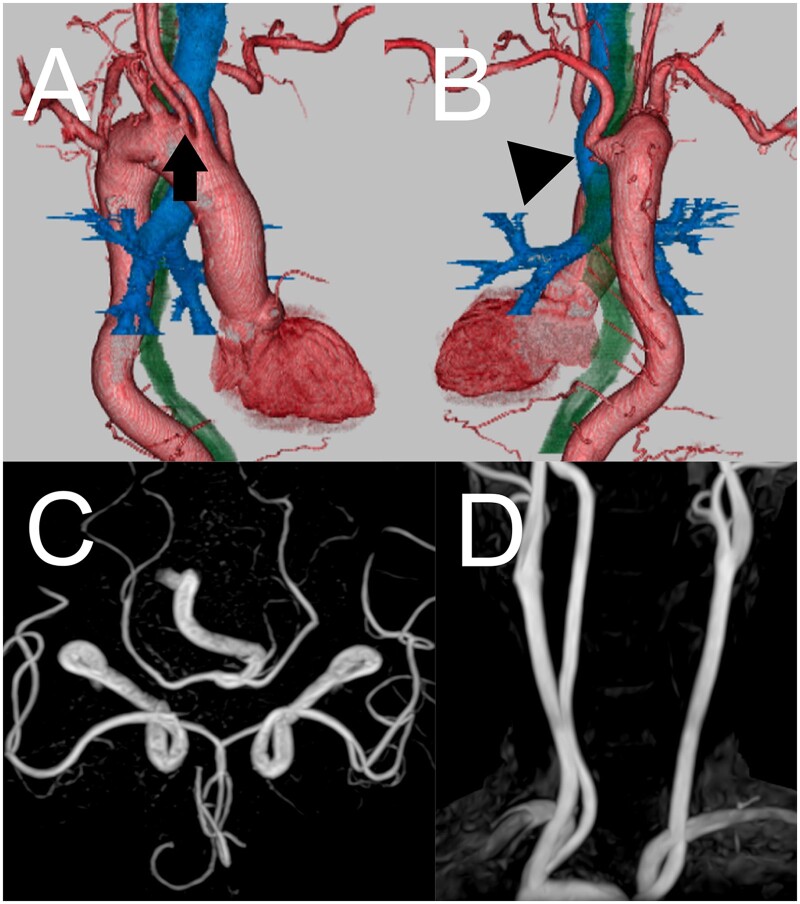
Preoperative three-dimensional computed tomography [(**A**) right lateral view; (**B**) posterior view] shows the steep right-sided aortic arch, the Kommerell diverticulum (black arrowhead) of the aberrant left subclavian artery and anomalous right vertebral artery originating from the aortic arch (black arrow). Preoperative magnetic resonance angiography shows the incomplete circle of Willis due to the absence of the left and right posterior communication arteries (**C**). Magnetic resonance angiography also reveals that the left vertebral artery is missing (**D**).

A 74-year-old man was referred after an incidental finding of an aortic arch anomaly. The patient was then referred to our hospital for a surgical consultation. He had a history of hypertension and hyperlipidaemia. He presented with an RAA with a KD of an ALSA arising from the descending aorta. The aortic arch was steep. The first branch of the arch was the left common carotid artery (LCA); the second vessel was the right common carotid artery (RCA); and the third vessel was the AVA. The fourth vessel was the right subclavian artery (RSA), and the fifth vessel was from the descending artery and was the ALSA. The ALSA passed behind the trachea and oesophagus, and an aortic diverticulum occurred from the origin of the ALSA, which is known as a Kommerell diverticulum (KD). The maximum distance from the opposite aortic wall to the tip of the KD was 51 mm. The left vertebral artery was missing (Fig. 1A and B). Additionally, magnetic resonance angiography revealed an incomplete circle of Willis due to the absence of the left and right posterior communication arteries (Fig. 1C and D). The AVA was the only artery to supply the vertebral-basilar system.

We planned a hybrid open arch procedure using a TAR with an FET. Further, we needed to reconnect the AVA due to the absence of the left vertebral artery and incomplete circle of Willis.

**Figure 2:**
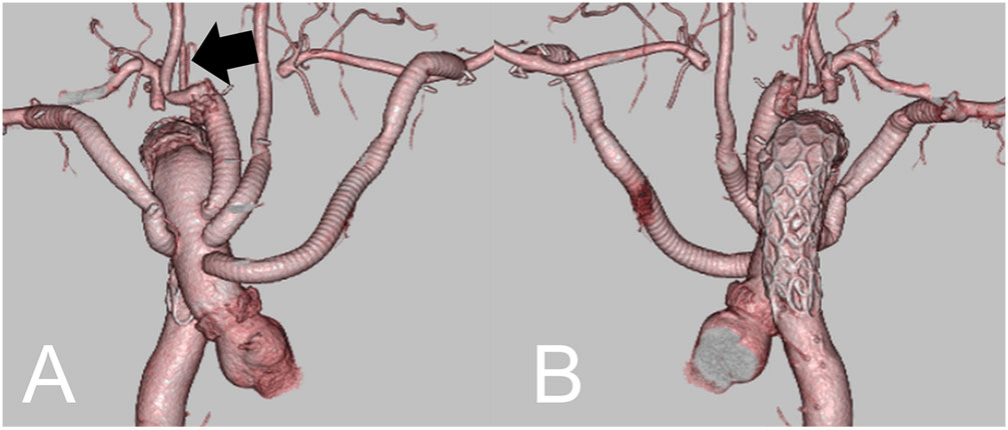
Postoperative three-dimensional computed tomography [(**A**) frontal view; (**B**) posterior view] shows good patency of the bypass graft and complete exclusion of the aneurysmal diverticulum. The black arrow points to the right vertebral artery.

Initially, a 9-mm tubular graft was anastomosed to both axillar arteries. Under moderate hypothermia and cardiopulmonary bypass, the aorta was transected between the AVA and RSA, and an FET (J graft FROZENIX 27 mm in diameter, 120 mm long, Japan Lifeline Co, Ltd, Tokyo, Japan) was inserted under selective cerebral perfusion. After deploying the FET, a stump of the graft was anastomosed to the 24 -mm four-branched graft. The lower body circulation was reinstituted through a branch graft. The proximal anastomosis was then completed, followed by coronary reperfusion. The 9-mm tubular graft from the left axillar artery was anastomosed to the first branch. The second branch (9 mm) was anastomosed to the LCA and the third branch (11 mm), to the AVA and the RCA in an island fashion. Finally, the 9-mm tubular graft from the right axillar artery was anastomosed to a sidearm branch (9 mm). The RSA was ligated at its origin. The ALSA was exposed just on the left side of the main bronchus and was ligated (Fig. 2).

His postoperative course was uneventful. A postoperative CT revealed good patency of the bypass graft and complete thrombosis of the KD. He was discharged 3 weeks after the surgery.

## DISCUSSION

An RAA is a rare congenital variant of the vascular anatomy with a prevalence of 0.05% to 0.1%. A KD has a high risk of an aortic dissection or rupture. It is generally accepted that one should consider a surgical intervention when the diameter of the diverticulum orifice exceeds 30 mm and/or the cross-sectional diameter from the opposite aortic wall to the tip of the diverticulum exceeds 50 mm [1–3].

Thus far, some reports have described the treatment of RAAs with KDs. Although it is unclear which treatment is the best, the current worldwide trend is towards endovascular procedures without a sternotomy. However, in our case, a simple thoracic endovascular aortic repair (TEVAR) with a coiling of the ALSA was not suitable because of the sharply curved arch and short landing zone. The length between the RSA and diverticulum was 17 mm. Also, a debranching TEVAR, consisting of an RCA-RSA bypass, an LCA-ALSA bypass and coil embolization to the ALSA, was unfit; that approach would not permit us to reconstruct the AVA, because the AVA was the only artery supplying the vertebral-basilar system.

According to Lars B *et al.*, 27.8% of both posterior communication arteries are missing [4]. Noticeably, however, the association between the incomplete Willis circle and only 1 vertebral artery changed the manner for handling the KD; we might have performed a debranching TEVAR, consisting of an RCA-RSA bypass and an LCA-ALSA bypass, if the patient had had either both sides of the vertebral artery or a normal circle of Willis. Therefore, it is important to perform preoperative magnetic resonance angiography in order to evaluate the anomalous artery.

On the other hand, a zone 0 landing TEVAR, consisting of an ascending aorta-LCA, RCA, AVA, RSA and LSA bypass with a coil embolization to the ALSA, could have been an alternative strategy. Likewise, a fenestrated repair might have been a feasible option. However, with such treatments there are some risks, such as a type 1 endoleak or migration of a stent graft over the long-term follow-up period.

A TAR with an FET could be the best treatment for RAAs with KDs associated with another arch vessel anomaly.
